# Supplementary material for: A Family Case of Congenital Myasthenic Syndrome-22 Induced by Different Combinations of Molecular Causes in Siblings
Source: Genes (Basel). 2020 Jul 19;11(7):821. doi: 10.3390/genes11070821 (PMC7397044; doi:10.3390/genes11070821)
Supplement: Supplementary file 1 [file genes-11-00821-s001.pdf]

Supplementary Materials:

Figure S1:

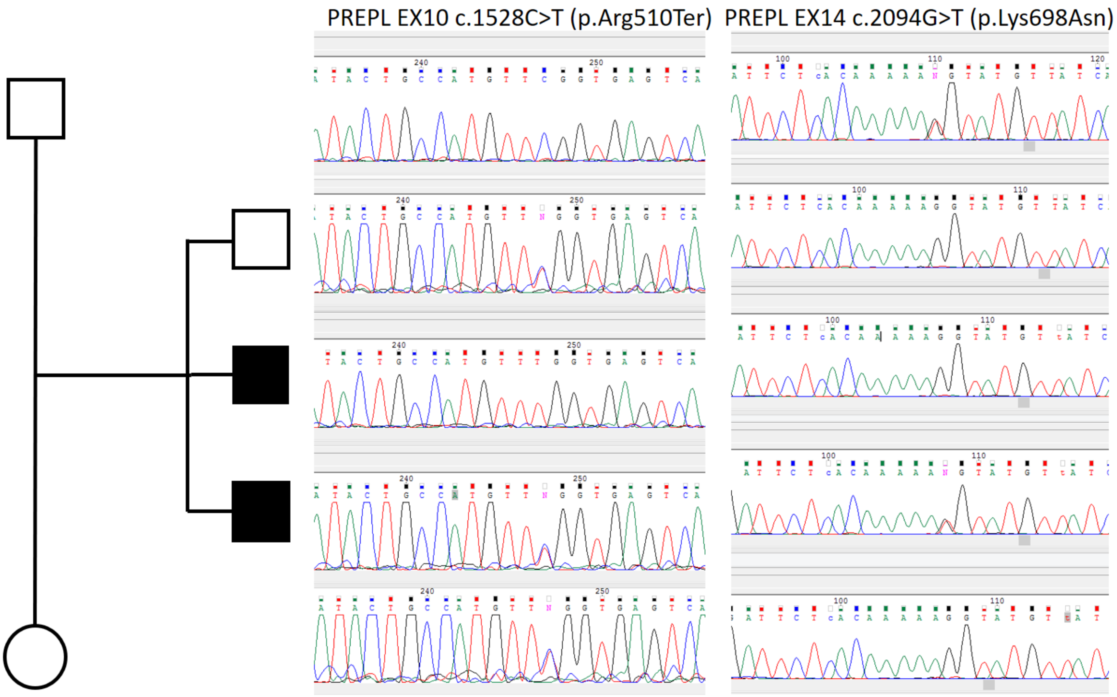

Sanger sequencing of *PREPL* exons 10 and 14.

Figure S2

		Probe target info	AllSamples							
			CYS 1.1	CYS 2.1	CYS 3.1	CYS 4.1	CYS 5.1	CYS 953	CYS 954	CYS 956
FRSS (n=7)		n/a	90%	100%	100%	100%	90%	90%	90%	90%
CAS (n=5)	FMRS	n/a	100%	100%	100%	100%	100%	100%	100%	100%
	PSLP - Relati...	n/a	OK	OK	OK	OK	OK	OK	OK	OK
	FSLP - Relati...	n/a	OK	OK	OK	OK	OK	OK	OK	OK
	RSQ - Refere...	n/a	Warning	Warning	Warning	Warning	Warning	Warning	Warning	Warning
	RPQ - Refere...	n/a	OK	OK	Warning	Warning	OK	OK	OK	OK
02p (n=24)	PPM1B-6	02-044,313084	1,02	1,1	1,06	1,24	0,98	0,95	0,97	0,93
	SLC3A1-1	02-044,356105	0,96	0,96	0,95	0,75	1,04	1,19	1,15	1,15
	SLC3A1-2	02-044,361489	0,92	1,15	0,78	0,96	1,17	1,05	1,11	0,93
	SLC3A1-3	02-044,362145	1,06	1,01	0,95	1	1,1	1	1	0,95
	SLC3A1-4	02-044,366525	0,92	0,73	1,02	1,03	0,86	1,05	1	1
	SLC3A1-5	02-044,380661	0,97	0,82	0,93	1	1,05	1,01	1	1,04
	SLC3A1-6	02-044,381780	0,97	1	0,9	0,93	1,06	1,06	1,01	1
	SLC3A1-7	02-044,384776	1	1,01	1,05	0,92	0,94	0,99	0,94	1,01
	SLC3A1-8	02-044,393290	0,93	0,97	1,08	0,91	0,93	0,96	0,93	1
	SLC3A1-9	02-044,394440	0,97	1,06	0,9	1,01	1,1	1,01	0,99	0,99
	SLC3A1-10	02-044,400839	1,05	1,02	1,09	1,04	0,96	0,88	0,95	0,91
	PREPL-15	02-044,401981	1,03	1,01	1,06	1,12	0,97	0,95	0,96	0,96
	PREPL-13	02-044,403245	0,95	0,96	0,98	0,9	1,03	1,02	1,05	1,06
	PREPL-12	02-044,403850	1	0,96	0,98	1,11	1,03	1	1	0,97
	PREPL-11	02-044,407402	1	0,92	0,97	0,97	1,01	1	1	1,04
	PREPL-10	02-044,409594	1,06	0,93	1,06	1,24	0,95	0,95	0,92	0,92
	PREPL-9	02-044,413101	1,02	1,01	0,98	1,08	1,07	0,87	0,88	0,81
	PREPL-7	02-044,419726	0,93	1,09	1,05	0,77	0,96	1,18	1,13	1,18
	PREPL-6	02-044,423103	1,01	1,06	1,03	0,99	0,97	0,99	1,01	0,98
	PREPL-5	02-044,424426	1,02	1,01	1,06	1,13	0,98	0,94	0,92	0,94
	PREPL-4	02-044,425188	0,88	0,91	0,97	1,01	1,03	1,1	1,15	1,05
	PREPL-2	02-044,440319	1,04	1,05	0,96	1,22	1,05	0,91	0,96	0,91
	PREPL-1	02-044,442205	1,01	1,11	0,98	0,88	1,11	0,99	1,04	1,03
	CAMKMT-2	02-044,453469	1	1,23	1,02	1,1	1	0,91	0,93	0,9
19q (n=15)	TDRD12-13	19-037,973452	1,04	1,04	0,96	1,12	1,04	0,93	0,93	0,93
	SLC7A9-13	19-038,013357	1,07	1,06	1,07	1,17	0,94	0,95	0,93	0,95
	SLC7A9-12	19-038,015951	0,99	0,9	1,02	1,02	0,94	0,99	1,02	1,02
	SLC7A9-11	19-038,024875	1,02	0,71	1,02	0,99	0,95	1	1	1,05
	SLC7A9-10	19-038,026617	1,02	1,1	1,01	1,09	0,98	0,98	0,95	0,98
	SLC7A9-9	19-038,041216	1,04	1,11	1,09	1,24	0,93	0,94	0,96	0,91
	SLC7A9-8	19-038,042576	1,01	0,93	1,05	1,07	0,95	1,02	0,96	0,99
	SLC7A9-7	19-038,043255	0,96	0,77	0,84	0,86	1,04	1,06	1,08	1,06
	SLC7A9-6	19-038,044903	0,96	1,04	0,91	0,77	1,05	1,24	1,16	1,24
	SLC7A9-5	19-038,045101	0,99	1,05	0,96	0,78	1,08	1,04	1,01	1,04
	SLC7A9-4	19-038,047010	0,98	1,08	1,02	0,82	0,95	1,15	1,12	1,19
	SLC7A9-3	19-038,047524	1,07	1,22	1,04	1,21	0,96	0,9	0,87	0,91
	SLC7A9-2	19-038,051169	1	1,04	1	0,99	1,01	0,98	0,98	1,01
	SLC7A9-1	19-038,052498	0,99	0,71	0,89	0,89	1,01	1,12	1,11	1,08
	CEP89-19	19-038,061925	1,05	1,29	1,07	1,05	0,95	0,87	0,91	0,92
References...	Reference*	01-214,565404	0,96	0,88	1,05	0,95	1	1,01	1	1,01
	Reference*	04-053,940141	0,97	1,04	0,89	0,9	1,04	1	1,02	1
	Reference*	07-075,448405	1,04	0,95	1,07	1,21	0,91	0,99	0,96	1,01
	Reference*	11-006,369211	1	1,17	0,88	0,97	1,05	1,02	1	0,95
	Reference*	12-013,797533	1,04	1,25	0,99	1,07	1,01	0,96	0,95	0,96
	Reference*	13-024,357452	0,95	0,91	0,9	0,94	1,06	1,23	1,21	1,16
	Reference*	16-055,463765	1	1,05	0,93	0,91	1	1,11	1,09	1,1
	Reference*	17-041,405030	1,04	0,92	1,11	1,1	0,97	1	1	1
	Reference*	18-045,817181	1,02	0,81	1,02	0,95	0,9	1,03	1,05	0,99

Results of quantitative analysis using SALSA MLPA Probemix P426 Cystinuria (MRC-Holland). 1.1 – Patient 1; 5.1 – Patient 2; 4.1 – healthy sibling; 3.1 – mother; 2.1 – father; CYS 953, CYS 954, CYS 956 - control samples of healthy adults

Figure S3

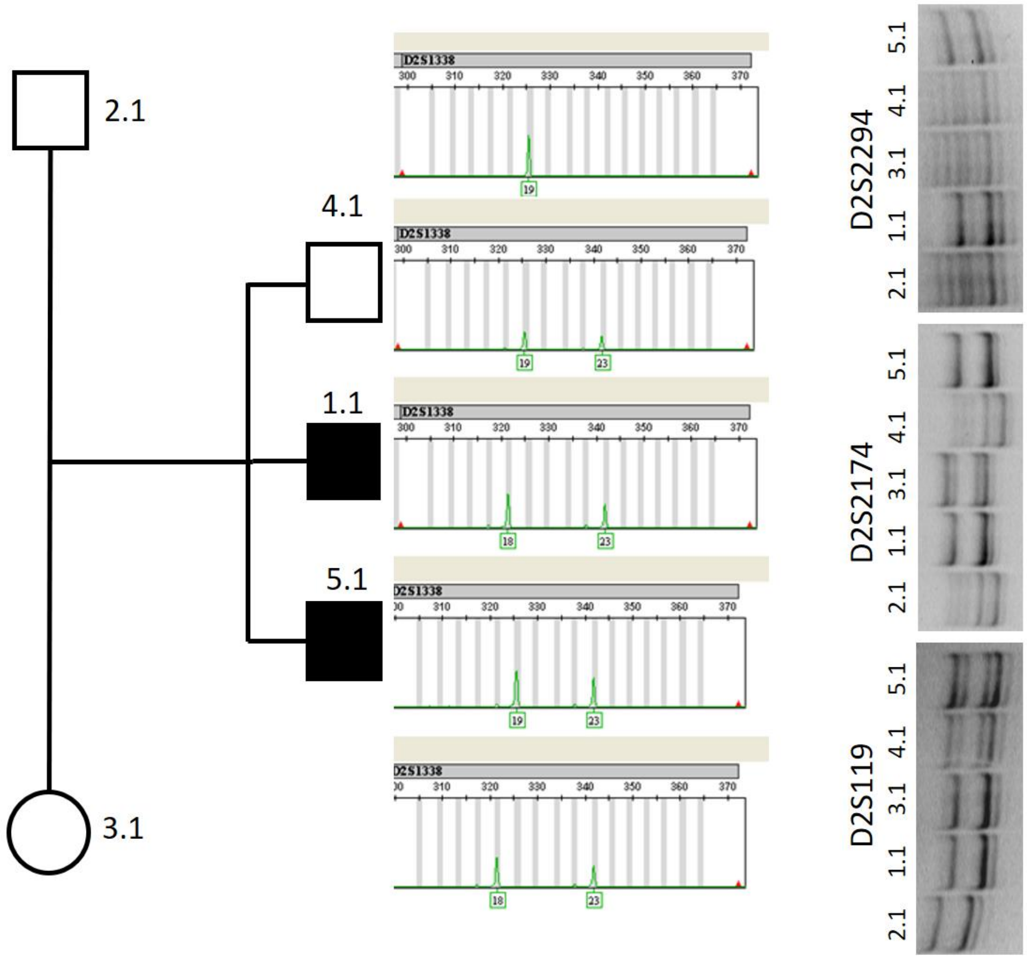

Genotyping results of chromosome 2 markers. 1.1 – Patient 1; 5.1 – Patient 2; 4.1 – healthy sibling; 3.1 – mother; 2.1 – father.
